# Effects of Relaxing Music on Mental Fatigue Induced by a Continuous Performance Task: Behavioral and ERPs Evidence

**DOI:** 10.1371/journal.pone.0136446

**Published:** 2015-08-25

**Authors:** Wei Guo, Jie Ren, Biye Wang, Qin Zhu

**Affiliations:** 1 School of Kinesiology, Shanghai University of Sport, Shanghai, China; 2 China Table Tennis College, Shanghai University of Sport, Shanghai, China; 3 Division of Kinesiology and Health, University of Wyoming, Laramie, Wyoming, United States of America; Catholic University of Sacro Cuore, ITALY

## Abstract

The purpose of this study was to investigate whether listening to relaxing music would help reduce mental fatigue and to maintain performance after a continuous performance task. The experiment involved two fatigue evaluation phases carried out before and after a fatigue inducing phase. A 1-hour AX-continuous performance test was used to induce mental fatigue in the fatigue-inducing phase, and participants’ subjective evaluation on the mental fatigue, as well as their neurobehavioral performance in a Go/NoGo task, were measured before and after the fatigue-inducing phase. A total of 36 undergraduate students (18–22 years) participated in the study and were randomly assigned to the music group and control group. The music group performed the fatigue-inducing task while listening to relaxing music, and the control group performed the same task without any music. Our results revealed that after the fatigue-inducing phase, (a) the music group demonstrated significantly less mental fatigue than control group, (b) reaction time significantly increased for the control group but not for the music group, (c) larger Go-P3 and NoGo-P3 amplitudes were observed in the music group, although larger NoGo-N2 amplitudes were detected for both groups. These results combined to suggest that listening to relaxing music alleviated the mental fatigue associated with performing an enduring cognitive-motor task.

## Introduction

Mental fatigue is a complex concept relevant to different areas of studies including physiology, sports medicine, psychology and therapy. In the present study, mental fatigue is defined as the feeling that people may experience during and following prolonged periods of cognitive activity requiring sustained mental activity [1]. Mental fatigue, which has been found to considerably disrupt task performance, is very common in daily life. It can lead to a variety of negative consequences, such as low productivity, poor study efficiency and even traffic accidents [2–5]. Thus, it is of great importance to explore the mechanisms underlying mental fatigue, and develop efficient methods to alleviate mental fatigue.

The effects of mental fatigue on cognitive-motor performance are well documented. Mental fatigue leads to decreased attention, delayed reaction times and increased number of errors during visual reaction tasks as a result of decreased levels of brain activation [1, 6–10]. Subjective feelings of mental fatigue are typically assessed by the Visual Analogue Scale, which asks participants to rate their subjective feelings of mental fatigue from 0 (minimum) to 100 (maximum) [11].

Using the Go/NoGo paradigm, increased mental fatigue has been found to increase the latencies of Go- and NoGo-P3 and NoGo-N2, while decreasing the amplitude of NoGo-P3 [1]. The amplitude of Go-P3, a late positive event-related potential (ERP) component, is an index of the amount of resources devoted to identifying a target stimulus. In addition, Go-P3 latency has been suggested as a measure of stimulus evaluation duration that is independent of response selection and execution processes [12–15].The NoGo-N2 component reflects detection of response conflict, indicated by an increased amplitude in high-conflict trials [16, 17].

A number of methods have been developed that alleviate the effects of mental fatigue on cognitive motor performance. First, attempts have been made to prevent mental fatigue before it occurs. For instance, ingestion of nutrients (such as a vitamin/mineral supplement) before a sustained cognitive task reduced subjective mental fatigue and improved the subsequent cognitive performance [18, 19]. Second, methods have been created to alleviate concurrent mental fatigue. It has been reported that the presence of intermittent odours during the completion reduced mental fatigue and improved attentional/effortful control of response selection, resulting in a better cognitive-motor performance [20]. Furthermore, Kaplan [21] reported that exposure to natural settings and stimuli such as landscapes and animals to reduce mental fatigue. In conclusion, interventions have been designed to either prevent or reduce mental fatigue. The focus of the present study was testing an intervention designed to reduce concurrent mental fatigue.

People tend to listen to music while working, studying or driving in daily life. It is believed that music may help alleviate mental fatigue and improve work efficiency. A number of studies have found that music affects people's cognitive processes [22–24]. For instance, Arikan et al. [22] reported that hearing music that was well-known by participants increased the allocation of attentional resources during memory updating processes in an auditory oddball task. This should also be reflected by a larger P3 amplitude. Also, it has been shown that listening to relaxing music is an effective way to recover from exercise-induced physical fatigue [25, 26]. Jing et al. [26] found that compared to those who had 15-minute rest without music after completing an exhausting cycle ergometer test, participants who listened to relaxing music for 15 minutes had a greater decrease in heart rate, urinary protein, and ratings of perceived exertion (RPE). However, the effect of listening to the relaxing music on the mental fatigue induced by the enduring cognitive motor task remained unknown. The present study was designed to examine this issue, along with the associated neurobehavioral mechanisms.

The Go/NoGo paradigm was used to examine the availability of participants’ attentional resources and conflict monitoring and inhibition process (as indexed by N2 and P3) following completion of the continuous performance task, with and without the presence of relaxing music. This will provide information in regards to the underlying neurobehavioral mechanisms. It was hypothesized that listening to relaxing music would result in a reduced subjective feeling of mental fatigue and reduce the negative effects of fatigue on cognitive-motor performance.

## Materials and Method

### Participants

36 undergraduate students (18 females and 18 males) between 18 and 22 years of age (M = 20.3) participated in the experiment. They were all right-handed, had normal or corrected-to-normal vision and normal audition. None of them was over-weight or obese (body mass index (BMI) of all participants was less than 25). None had a history of current or past neurological or psychiatric illnesses and none was taking any medication known to affect the central nervous system. Table 1 shows the main characteristics of the participants.18 males and 18 females were randomly assigned to the control group and the music group. Both groups performed the same tasks (fatigue-inducing and fatigue-evaluating tasks), however, the control group performed the 60-min fatigue inducing task in a quiet condition, and the music group performed the same task while listening to relaxing music. Written informed consent was obtained from all participants prior to the study. Participants didn’t know the real purpose of the study and were paid for their participation. The Ethical Committee of Shanghai University of Sport approved the manner of consent and the study.

**Table 1:** Main characteristics of the participants.

	total	music group	control group
Age(yr)	20.33 ± 1.26	20.31 ± 1.25	20.35 ± 1.31
Height(m)	1.68 ± 0.09	1.66 ±0.08	1.69 ± 0.10
Weight(kg)	60.43 ± 10.75	59.72 ± 10.31	61.00 ± 11.32
BMI(kg/m^2^)	21.24 ± 2.14	21.34 ± 2.60	21.16 ± 1.75

### Procedures

Participants were instructed to abstain from alcohol 24 h before the experiment and from caffeine-containing substances 12 h before the experiment. Each participant was seated in a dimly lit, electrically shielded room with response keyboard in his/her right hand. The experiment consisted of 3 continuous phases. The first phase was filled with a 15min long Go/NoGo task as fatigue-evaluating task. Participants could have a rest every 5min in order to prevent fatigue. After which, participants were required to fill out the VAS. Phase 2 was filled with the fatigue-inducing task, during which participants performed 60 min AX-continuous performance task without rest. Then participants performed the VAS again and finished phase 3 with another 15min long fatigue-evaluating task, which was the same as the first phase. The participants were instructed to respond as quickly as possible, maintaining a high level of accuracy. Both the control and music group performed the fatigue-evaluating task with the concurrent electroencephalography (EEG) data recorded, which was not performed during the fatigue-inducing phase. While performing the fatigue-inducing task, participants in the music group listened to the relaxing music, those in the control group did not. The relaxing music was chosen from functional music developed by the China Institute of Sport Science. These pieces of music helped Chinese athletes alleviate mental fatigue during the preparing for the 2008 Beijing Olympic Games. All participants listened to the same 12 pieces of instrumental folk music (no lyrics) music during the fatigue-inducing phase. The chosen pieces were played to the participants at a tempo of 60–80 beats per minute. The music was presented via a power amplifier connected to a computer, with a volume of 40dB.

#### Fatigue-evaluating task

**Fig 1:**
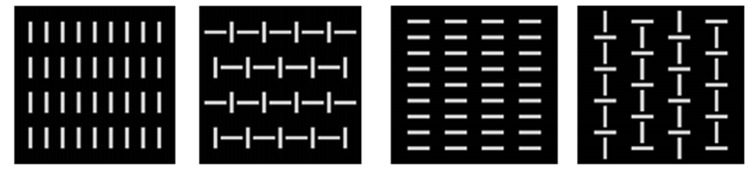
Stimuli used in Go (the two pictures on the right) and NoGo (the two pictures on the left) task.

A Go/NoGo paradigm was used to measure effect of mental fatigue. A computer screen (19inch) was positioned approximately 1m in front of the participants. The fixation point was a cross (1×1° of visual angle) displayed in the center of the screen for 1500ms. Four squared configurations subtending 4×4° were presented for 100ms on a dark background, and were displayed randomly with equal probability (p = 0.25), and a stimulus onset time of 800ms. Two configurations were defined as targets and two non-targets (Fig 1). The participants were instructed to press a button as quickly as possible with their right hand when the target appeared on the screen (Go stimuli; p = 0.5), and withhold responding when a non-target appeared (NoGo stimuli; p = 0.5). The task consisted of 4 blocks of 400 trials.

#### Fatigue-inducing task

Mental fatigue was induced by asking participants to work on the AX-continuous performance task for 60 min. In this task, letters that consisted of cue stimuli (target letter A and non-target letter B) and probe stimuli (target letter X and non-target letter Y) were presented sequentially on a computer screen. Participants sat in front of a numeric keyboard and were instructed to give a target response (press button’1’ on a numeric keyboard) to a probe letter ‘X’ after a cue letter ‘A’. In all other cases, they had to respond with a non-target response (button’3’ on the same keyboard). The frequency of A-X trails was 70% and A-Y, B-X, B-Y trails were 10% respectively.

#### Subjective evaluation of Mental Fatigue (VAS)

The participants were asked to subjectively rate their alert/concentrated, anxious, energetic, confident, irritable, jittery/nervous, sleepy, talkative levels on a visual analogue scale (VAS) from 0 (minimum) to 100 (maximum). Previous studies have shown that the VAS was valid for subjective evaluation of mental fatigue [11, 27]. Participants were asked to perform ratings before and after the fatigue-inducing phase in order to assess the change in subjective feelings after performing the task trials.

### Data recording and analysis

Reaction time and error rate were measured as behavioral variables via e-prime (Psychology Software Tools, Pittsburgh, PA, USA). With the 10–20 system, the electroencephalogram (EEG) signals were recorded from 64 electrodes attached to an electrocap (Brain Products, BrainAmp MR Plus), and all electrodes were referenced to linked ears. A ground electrode located between Fpz and Fz. The electro-oculogram (EOG) was recorded bipolarly from two electrodes placed at the outer canthi of the right eye and below the left eye. Electrode impedance was kept below 5 kΩ. Signals were digitized at a rate of 1000 Hz.

ERPs were collected with the Brain Products recorder software and were analyzed off-line by its analyzer. The ERP signals were averaged off-line for the correct responses only. Trails contaminated with artifacts greater than ±120μV were rejected before averaging and the averaged ERPs were smoothed through a low-pass digital filter at 30 Hz. The epoch for ERP analysis was 1000 ms (200 ms before and 800 ms after the stimulus onset). For ERP analysis, 3 electrodes of Fz, Cz and Pz were selected. The ERP components were specifically described by N2 (range 200–350 ms) and P3 (range 350–600 ms) in both Go and NoGo trails during the fatigue-evaluating task.

### Statistical analysis

Statistical analyses of subjective data, behavioral data and ERP data were performed using a mixed-design ANOVA. The between-subjects variable was condition (music and control), and the within-subject variable was the testing phase (before and after fatigue-inducing task). Degrees of freedom were corrected whenever necessary using the Greenhouse-Geisser epsilon correction factor. The significant level was set at p <0.05.

## Results

### Fatigue Scale

The VAS scores on anxious (F_(1,34)_ = 19.13, p <0.01, η_p_^2^ = 0.36), irritable (F_(1,34)_ = 8.21, p <0.01, η_p_^2^ = 0.20), sleepy (F_(1,34)_ = 21.81, p <0.01, η_p_^2^ = 0.39) significantly increased; and the scores on alert/concentrated (F_(1,34)_ = 50.69, p <0.01, η_p_^2^ = 0.60), energetic (F_(1,34)_ = 73.47, p <0.01, η_p_^2^ = 0.68), confident (F_(1,34)_ = 42.43, p <0.01, η_p_^2^ = 0.56) significantly decreased after the fatigue-inducing mental task session. There was also a significant interaction between testing phase and condition for alert/concentrated (F_(1,34)_ = 14.69, p <0.01, η_p_^2^ = 0.30), anxious (F_(1,34)_ = 9.09, p <0.01, η_p_^2^ = 0.21), energetic (F_(1,34)_ = 12.52, p <0.01, η_p_^2^ = 0.27), confident (F_(1,34)_ = 10.04, p <0.01, η_p_^2^ = 0.23) and irritable (F_(1,34)_ = 6.29, p <0.05, η_p_^2^ = 0.16). Further interaction contrasts for different testing time showed that before the fatigue-inducing phase all the subjective levels were not significantly different between the control and music groups. However, after the fatigue-inducing phase, the music group’s scores on alert/concentrated (F_(1,34)_ = 12.08, p <0.01), energetic (F_(1,34)_ = 8.79, p <0.01), confident (F_(1,34)_ = 11.35, p <0.01) were significantly higher than those of control group, and the control group’s scores on anxious (F_(1.34)_ = 5.63, p <0.05) and irritable (F_(1,34)_ = 5.81, p <0.05) were significantly higher (see Table 2 and S1 Table for supplement). These results suggested that our fatigue-inducing task did result in mental fatigue on both groups, but it affected control group more than music group.

**Table 2:** Scores of the fatigue scale.

	Before task		After task	
	Control group	Music group	Control group	Music group
alert/concentrated	73.8 ± 12.89	72.22 ± 16.29	35.00 ± 22.82	60.55 ± 21.27**
anxious	26.11 ± 16.14	27.78 ± 18.96	53.33 ± 30.29	32.78 ± 20.81*
energetic	72.22 ± 14.37	67.78 ± 16.29	29.44 ± 18.93	50.00 ± 22.49**
feel confident	72.22 ± 15.17	73.33 ± 15.72	41.67 ± 18.86	62.78 ± 18.73**
irritable	22.22 ± 23.90	20.56 ± 14.74	38.89 ± 24.71	21.67 ± 17.57*
jittery/nervous	23.89 ± 22.00	25.00 ± 18.86	31.67 ± 23.57	26.11 ± 19.44
sleepy	41.67 ± 25.26	43.33 ± 26.57	72.78 ± 22.70	57.22 ± 20.52
talkative	32.78 ± 24.21	25.00 ± 16.89	27.22 ± 21.64	22.78 ± 13.20

** p <0.01

*p <0.05

### Behavioral performance

**Fig 2:**
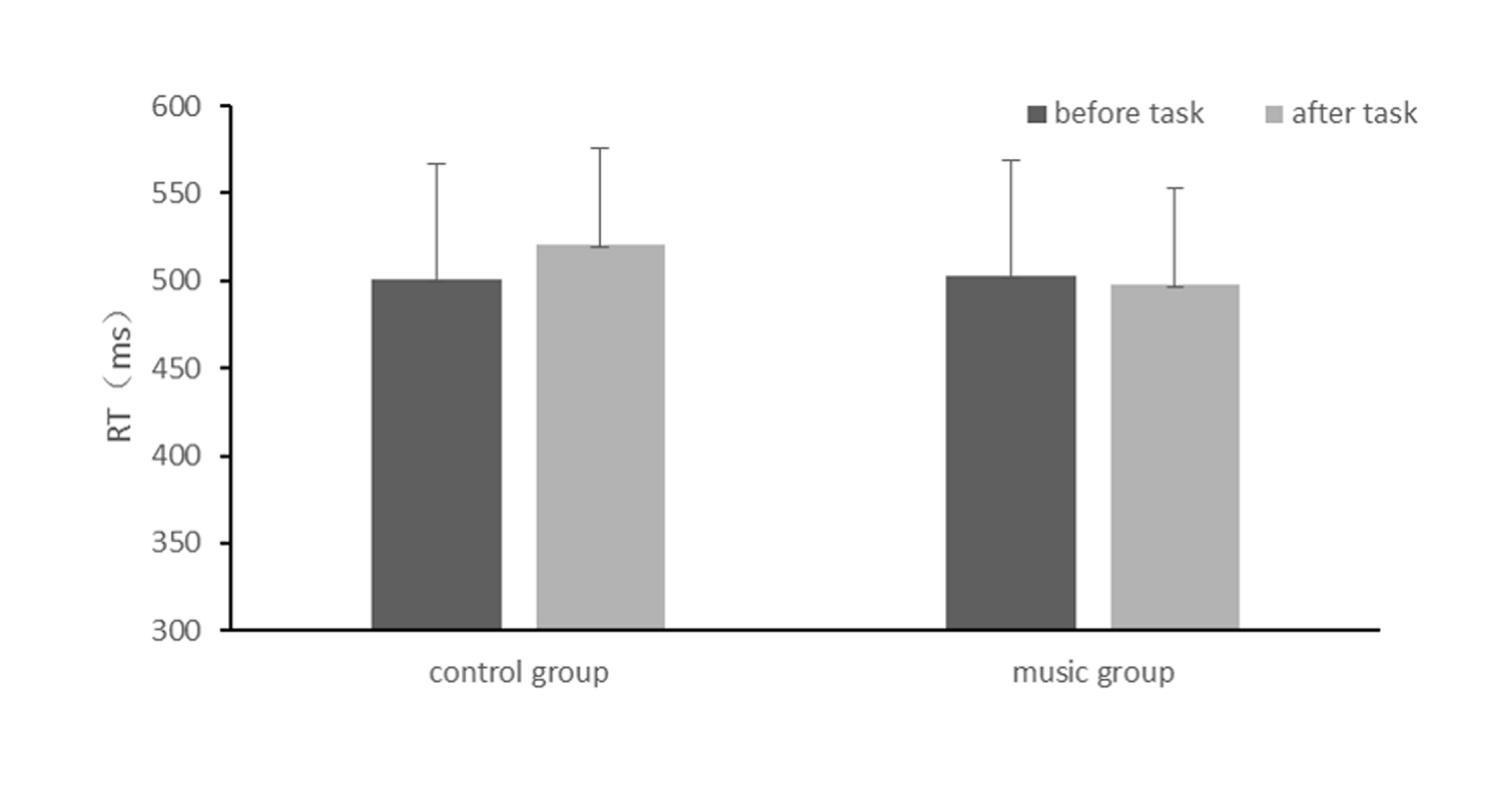
Average RTs for Go trials as a function of condition and testing time.

Reaction times of Go trials significantly increased after the fatigue-inducing task (F_(1,34)_ = 3.98, p = 0.05, η_p_^2^ = 0.11). There was a significant interaction between condition and test phase (F_(1,34)_ = 10.85, p <0.01, η_p_^2^ = 0.24). An interaction contrast indicated that reaction time significantly increased for the control group (500ms to 520ms), but remained the same for the music group (502ms to 498ms). The accuracy of the task did not differ at all (see Fig 2 and S2 Table for supplement).

### P3 component

**Fig 3:**
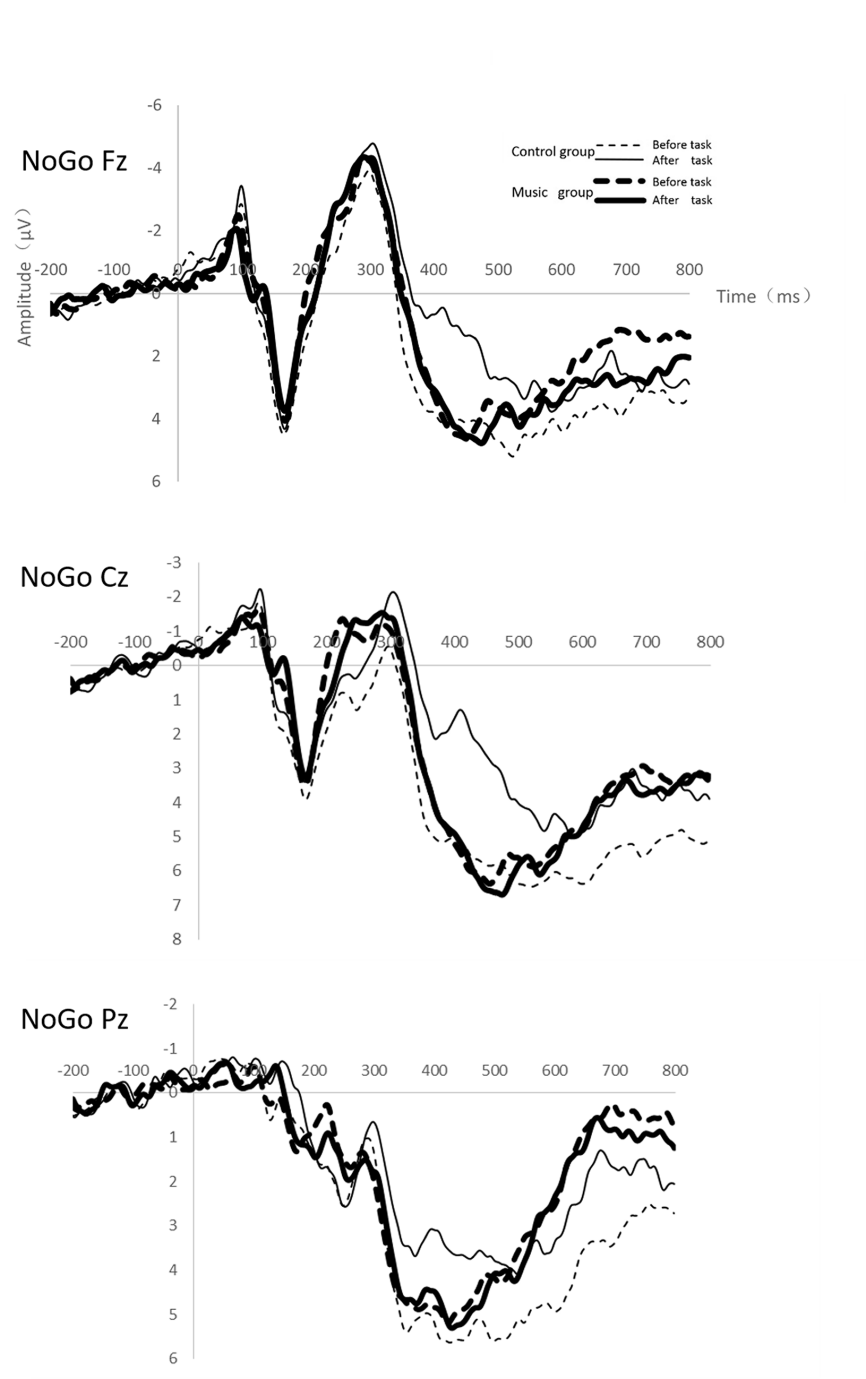
Grand-average ERPs at the Fz, Cz and Pz sites for NoGo trials as a function of condition and testing time.

Fig 3 shows the grand-average ERPs at the Fz, Cz and Pz sites for NoGo trails. The NoGo-P3 amplitudes at Cz and Pz sites significantly decreased after the fatigue-inducing phase (F_(1,34)_ = 9.64, p <0.01, η_p_^2^ = 0.22; F_(1,34)_ = 4.63, p = 0.04, η_p_^2^ = 0.12, respectively). The interactions between testing phase and condition were significant at the Cz and Pz sites (F_(1,34)_ = 8.95, p <0.01, η_p_^2^ = 0.21; F_(1,34)_ = 4.91, p = 0.03, η_p_^2^ = 0.13, respectively). An interaction contrast at the Cz and Pz sites indicated that the NoGo-P3 amplitudes were significantly different on testing phase for the control group (F_(1,34)_ = 6.04, p = 0.02, F_(1,34)_ = 4.33, p = 0.05, Cz 6.65 mV, 1.87 mV; Pz 5.53 mV, 3.46 mV), but they were not significant for the music group. Although the NoGo-P3 latencies at the Fz, Cz and Pz sites all increased after the fatigue-inducing task, there was no significant difference between the groups.

**Fig 4:**
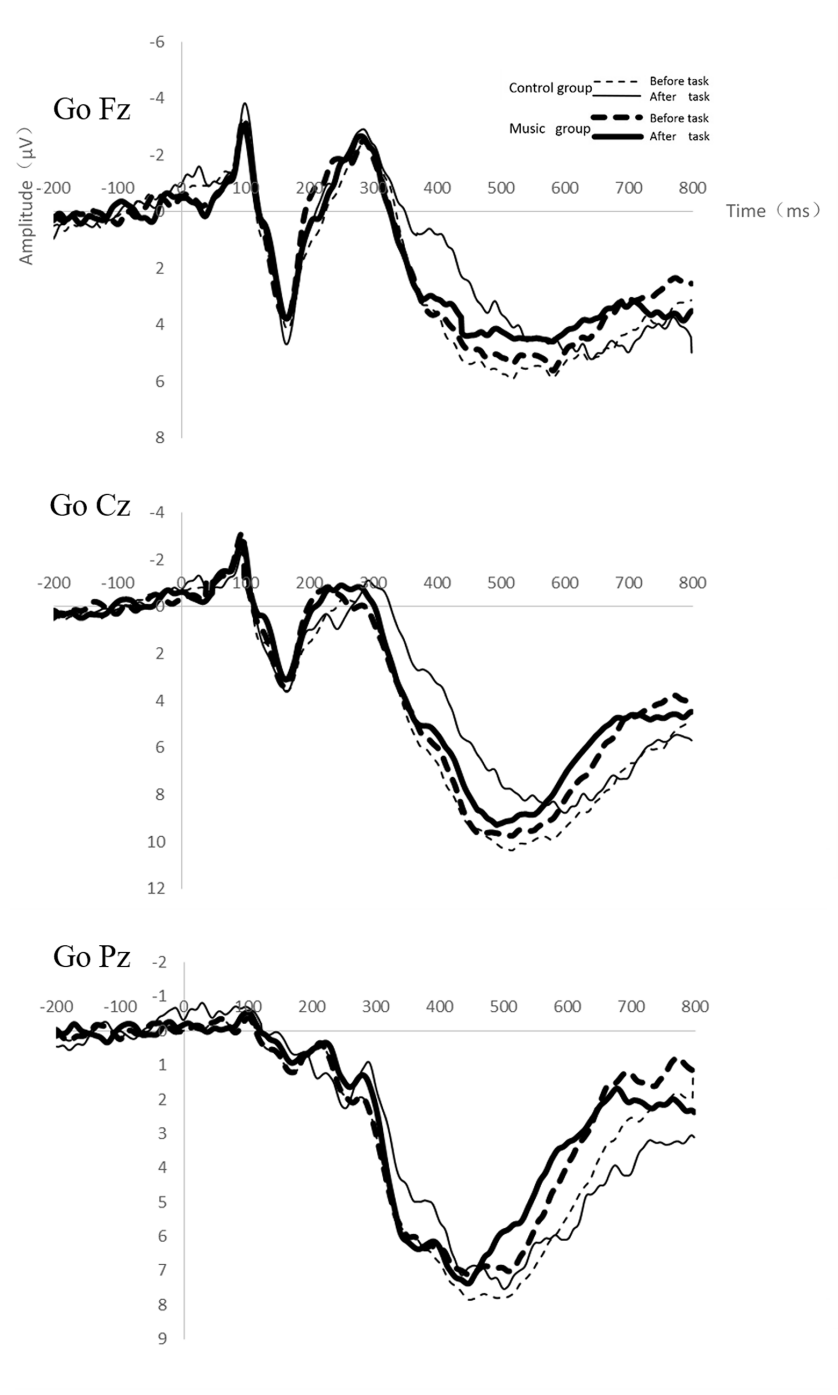
Grand-average ERPs at the Fz, Cz and Pz sites for Go trials as a function of condition and testing time.

Fig 4 shows the grand-average ERPs at Fz, Cz and Pz for Go trials as a function of condition and time-on-task. The Go-P3 amplitudes at Fz and Cz sites significantly decreased after the fatigue-inducing task (F_(1,34)_ = 4.70, p = 0.04, η_p_^2^ = 0.12; F_(1,34)_ = 4.81, p = 0.04, η_p_^2^ = 0.12); The interaction between testing phase and condition at Cz sites was significant (F_(1,34)_ = 4.44, p = 0.04, η_p_^2^ = 0.12); An interaction contrast at the Cz site indicated that the Go-P3 amplitude of the control group was smaller than that of the music group (4.52 mV, 6.16 mV) after the fatigue-inducing task. No main effect of condition was found. The Go-P3 latencies at the Fz, Cz, Pz sites all increased after the fatigue-inducing phase, but the main effect and interaction were not significant (See S3 Table for description of ERP data).

### N2 component

For the NoGo-N2 amplitudes, the main effect of testing phase was significant at the Cz site (F_(1,34)_ = 4.35, p = 0.05, η_p_^2^ = 0.11).There were no other main effects or interactions found. It revealed that although NoGo-N2 amplitudes increased at the Cz site after the fatigue-inducing phase, there were no significant differences between the groups (Fig 3).

As for the Go-N2 amplitudes at Fz, Cz and Pz, the main effects of testing phase and condition, as well as their interaction were not significant (Fig 4).

## Discussion

In the present study, both the music group and control group exhibited signs of mental fatigue (distracted, anxious, irritable, sleepy and unconfident) after the 60-min of fatigue-inducing task. This is in accord with previous findings [28] and suggests that our task manipulation was successful in inducing mental fatigue. Importantly, based on the scores of Visual Analogue Scale, the control group reported suffering from more mental fatigue than the music group.

This phenomenon was also demonstrated by behavioral data. After the fatigue-inducing phase, the reaction time of the control group increased on the Go/NoGo task, while the music group remained the same. The accuracy of the two groups did not change in the Go/NoGo task, which could be due to the flooring effect, where participants had maintained a fairly high accuracy in the task. The behavioral results implied that when people perform cognitively challenging tasks, listening to relaxing music could mitigate performance deterioration (e.g., a slower response) caused by mental fatigue.

The observed performance deterioration might be related to a reduction in attentional resources allocated to the task due to mental fatigue. Kok [29] suggested that the attentional availability of resources is the most important factor in determining the performance in the cognitive task. Lack of attentional resources will lead to the poorer performance. In our study, the Go-P3 amplitude significantly decreased after the fatigue-inducing task for the control group, suggesting that participants had increasing difficulty allocating resources for stimulus detection as the task unfolded. In Kato’s study, they found that mental fatigue had no effect on the Go-P3 amplitude[1], which was inconsistent with ours. We assumed that different proportion of the Go and NoGo trials used for study would account for this difference. In their study, the stimulus probability of Go trials (80%) was greater than that of NoGo trials (20%). As mental fatigue may cause the decreased availability of cognitive resources [8, 20], in order to maintain a speedy response, the most reasonable decision is to allocate the limited resources to the Go stimuli instead of the NoGo stimuli. Therefore, participants may have limited capacity to allocate attentional resources to the NoGo stimulus alone due to mental fatigue. The stimulus probability of Go trails and NoGo trials in our study was 50% to 50%, thus, participants detected the two stimuli with equal probability, and they had to allocate equal amount of attentional resources to both Go and NoGo stimuli. Interestingly, the Go-P3 amplitude decreased less in the music condition compared to the control condition after the fatigue-inducing task, suggesting that the attentional availability of resources did not decline much in the music condition. Hence, participants who performed the fatigue-inducing task in the music condition suffered less mental fatigue.

N2 is an important ERP component in NoGo trails. Many studies have been performed to clarify the cognitive process reflected by NoGo-N2, some of which suggested that NoGo N2 reflected conflict monitoring[30, 31], and others thought it reflected response inhibition[32–34]. More and more evidence suggested that N2 might reflect the conflict monitoring process. Bruin et al. [35] examined the effects of response priming on N2 evoked by target stimuli in a Go/NoGo task. They found that N2 was not modulated by response priming, and concluded that it was not associated with response inhibition. Instead they proposed the frontal P3 to be inhibition-specific. This positive wave peaked between 300 and 600 ms after stimulus presentation and was enhanced on NoGo trials relative to Go trials on the frontal electrode channels. In the present study, we found that the decrement of NoGo-P3 amplitudes after the fatigue-inducing task were smaller in the music condition than in their absence. These findings suggest that listening to the relaxing music plays an important role in mitigating the deterioration of cognition performance and inhibition resulted from mental fatigue. For the NoGo-N2 trials, there was only a main effect of testing phase at Cz site with no group difference. We assumed that music only modulated response inhibition without effect on the conflict monitoring process. The relationship between mental fatigue and response inhibition remains unclear. Falkenstein et al. [36] investigated the inhibition-related ERP components, and they found that there was no effect of time on task on NoGo-P3 component, therefore, they suggested that the inhibitory processes were fairly robust against mental fatigue. In Kato’s study [1], the NoGo-P3 amplitude decreased with time on task, suggesting that mental fatigue affected controlled processing for response inhibition. At this point, our findings were consistent with Kato’s. The NoGo-P3 amplitudes at Cz and Pz sites significantly decreased after the fatigue-inducing task, indicating the response inhibition was impaired. These findings combined to suggest that listening to the relaxing music mitigated the decrement of response inhibition during mental fatigue.

A previous study found that the presence of intermittent odours during the continuous performance task helped to reduce mental fatigue and maintain performance [20]. The present study showed the similar effect using relaxing music. Listening to the relaxing music during a cognitive task may make people feel less mentally fatigued and maintain high productivity. The effects of relaxing music on alleviating mental fatigue may better explain why people tend to listen to music while working, studying or driving in daily life. Nevertheless, there are some limitations of the study. Firstly, the fatigue-inducing task AX-CPT is a relatively simple task, and so future research should attempt to replicate results using a more difficult task to induce mental fatigue. Secondly, the relaxing music might act as a distracting stimulus, blocking mental fatigue instead of exerting a relaxing effect. A group listening to a non-musical sound may be added as a better control in the future experiment.

## Conclusion

The current study suggests a potential intervention for reduction in mental fatigue while performing an enduring cognitive-motor task. By listening to relaxing music during a fatigue-inducing task, both mental fatigue and deterioration in motor performance were reduced. The analysis of ERP signals further suggested that the music group suffered less impairment of attentional control of response selection and inhibition than the control group. In sum, these findings demonstrate that listening to relaxing music can alleviate the mental fatigue encountered in performing an enduring cognitive-motor task at both behavioral and cognitive levels.

## Supporting Information

S1 Table Fatigue scale data. (XLSX)

S2 Table Behavioral data. (XLSX)

S3 Table ERP data. (XLSX)
